# Mixed responses to first‐line alectinib in non‐small cell lung cancer patients with rare *ALK* gene fusions: A case series and literature review

**DOI:** 10.1111/jcmm.16897

**Published:** 2021-09-19

**Authors:** Mengnan Li, Zhou An, Qiusu Tang, Yutong Ma, Junrong Yan, Songan Chen, Yina Wang

**Affiliations:** ^1^ Department of Medical Oncology First Affiliated Hospital College of Medicine Zhejiang University Hangzhou China; ^2^ Department of Thoracic Surgery First Affiliated Hospital College of Medicine Zhejiang University Hangzhou China; ^3^ Department of Pathology First Affiliated Hospital College of Medicine Zhejiang University Hangzhou China; ^4^ Nanjing Geneseeq Technology Inc. Nanjing China; ^5^ Burning Rock Biotech Guangzhou China

**Keywords:** alectinib, *LMO7‐ALK*, NSCLC, rare *ALK* fusion, *STRN‐ALK*, survival

## Abstract

Anaplastic lymphoma kinase (*ALK*) fusion is a well‐defined biomarker for ALK tyrosine kinase inhibitors (TKIs) treatment in non‐small cell lung cancer (NSCLC). Alectinib, a second‐generation ALK‐TKI, has been shown to have significantly longer progression‐free survival (PFS) than first‐generation ALK inhibitors in untreated *ALK*‐rearranged NSCLC patients. However, its clinical efficacy on rare *ALK* fusions remains unclear. Herein, two advanced NSCLC patients received first‐line alectinib treatment, given their positive *ALK* fusion status as determined by immunohistochemistry (IHC) testing results. Patients showed limited clinical response (PFS: 4 months) and primary resistance to alectinib respectively. Molecular profiling using next‐generation sequencing (NGS) further revealed a striatin (STRN)‐ALK fusion in the first patient accompanied by *MET* amplification, and a LIM domain only protein 7 (LMO7)‐ALK fusion in another patient without any other known oncogenic alterations. Both patients demonstrated improved survival after they switched to second‐line crizotinib (PFS: 11 months) and ensartinib (PFS: 18 months), respectively, up till the last follow‐up assessment. In conclusion, the clinical efficacy of ALK‐TKIs including alectinib for lung cancer with uncommon *ALK* gene fusions is still under evaluation. This study and literature review results showed mixed responses to alectinib in NSCLC patients who harboured rare *ALK* fusions. Comprehensive molecular profiling of tumour is thus strongly warranted for precise treatment strategies.

## 1 INTRODUCTION

Anaplastic lymphoma kinase (*ALK*) fusion was first identified in 2007 with echinoderm microtubule associated protein like 4 (*EML4*) as it’s most common fusion partner gene. Alectinib is a second‐generation ALK tyrosine kinase inhibitor (TKI), which showed much better first‐line efficacy than first‐generation ALK‐TKI, crizotinib, with median progression‐free survival (PFS) being 34.8 months in untreated ALK‐positive non‐small cell lung cancer (NSCLC).^1^ However, the ALK status of patients enrolled in ALEX clinical trial was only confirmed by immunohistochemistry (IHC), while detailed information about *ALK* fusion partners was unclear. Thus, it remains to be further investigated whether alectinib demonstrates comparable efficacy in patients with different *ALK* fusion partners including rare *ALK* fusions. In this study, we described two NSCLC cases in which rare *ALK* fusions, striatin (*STRN*)‐*ALK* and LIM domain only protein 7 (*LMO7*)‐*ALK*, as identified by next‐generation sequencing (NGS), showed unfavourable responses to alectinib treatment.

## 2 CASE PRESENTATION

### 2.1 Case 1

**FIGURE 1:**
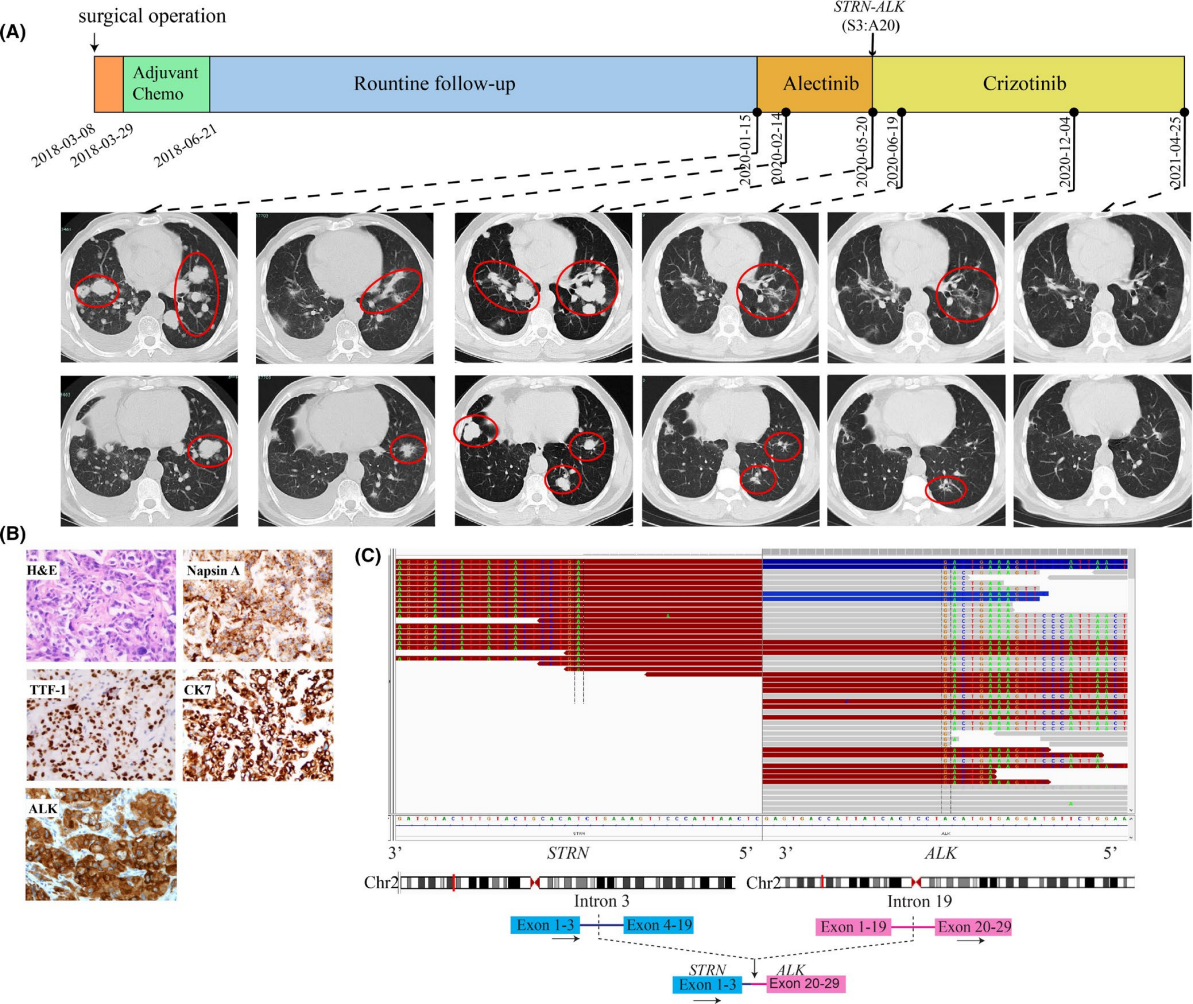
Schematic of treatment history and NGS‐detected *ALK* fusion of Case 1. (A) The timeline of treatment and CT scans are showed where the red circles highlight the location of tumours. The time point of surgery and NGS test are labelled above. (B) Pathological examination of the surgical specimen. IHC testing (400×) results showed positive expression of Napsin A, TTF‐1, CK7 and ALK. (C) Identification of the *STRN*‐*ALK* fusion. Sequencing reads of *ALK* and *STRN* are visualized by the Integrative Genomics Viewers (IGV, top panel). The schematic below shows the fused exons of the *STRN*‐*ALK* rearrangement

A 42‐year‐old Chinese male patient without smoking history was diagnosed as adenocarcinoma of right lung (pT2aN2M0, stage IIIA) and had received right middle lobe lobectomy, mediastinal lymph node dissection in March 2018 in a local hospital. He received pemetrexed plus cisplatin for 4 cycles as adjuvant chemotherapy and kept on following up with an average interval of 6 months. Then, the disease relapsed in January 2020 when right pleural effusion, multiple pulmonary metastases (maximum diameter: 28 mm) and mediastinal lymph enlargement were detected by routine follow‐up computed tomography (CT) scans in our hospital (Figure 1A). Haematoxylin and eosin (HE) staining of the primary surgically resected tumour sample revealed typical adenocarcinoma histology with positive IHC markers including Napsin A, cytokeratin 7 (CK7), thyroid transcription factor‐1 (TTF‐1) and ALK (Ventana, Figure 1B). Thus, alectinib (600 mg, twice a day) was prescribed since January 2020. The follow‐up chest CT scans after one month showed a partial response (PR) (maximum diameter: 18 mm) based on response evaluation criteria in solid tumours [RECIST] version 1.1. However, the disease progressed rapidly as the contrast CT scans performed in May 2020 showed multiple enlarged metastatic nodules in both lungs (maximum diameter: 41 mm, Figure 1A). NGS on CT‐guided lung puncture specimen revealed a rare *STRN*‐*ALK* fusion (Figure 1C) accompanied with *MET* amplification (Table 1). The patient was then switched to crizotinib treatment (250 mg, twice a day) starting from May 2020, and clinical response was achieved after one month when CT scans showed shrinkages of target nodules in both lungs (maximum diameter: 22 mm, Figure 1A). As the last follow‐up on 25 April 2021, the patient remained stable with a PFS for over 11 months.

**TABLE 1:** Concurrent alterations identified in patients

Case 1 (alteration, allele frequency)	Case 2 (alteration, allele frequency)
*STRN*:exon3~*ALK*:exon20 (25.9%)	*LMO7*:exon15~*ALK*:exon20 (15.3%)
*MET* amplification (3.8‐fold)	*NRG1*:c.602A>T (21.0%)
*TP53*:c.991C>T (6.3%)	*TP53*:c.314G>A (2.2%)
*TP53*:c.742C>7 (26.8%)	*TP53*:c.140del (1.4%)
*BRCA1*:c.5347A>C (4.6%)	*TP53*:c.637C>T (0.9%)
*BRCA2*:c.7835C>A (3.8%)	
*FANCM*:c.3296G>A (6.1%)	
*IDH1*:c.940C>T (4.2%)	
*LRP1B*:c.139T>C (3.3%)	
*MSH6*:c.1589A>G (2.8%)	
*NTRK2*:c.718_719del (5.3%)	
*PMS1*:c.2417C>G (5.1%)	
*PTPN13*:c.2516C>T (17.2%)	

### 2.2 Case 2

**FIGURE 2:**
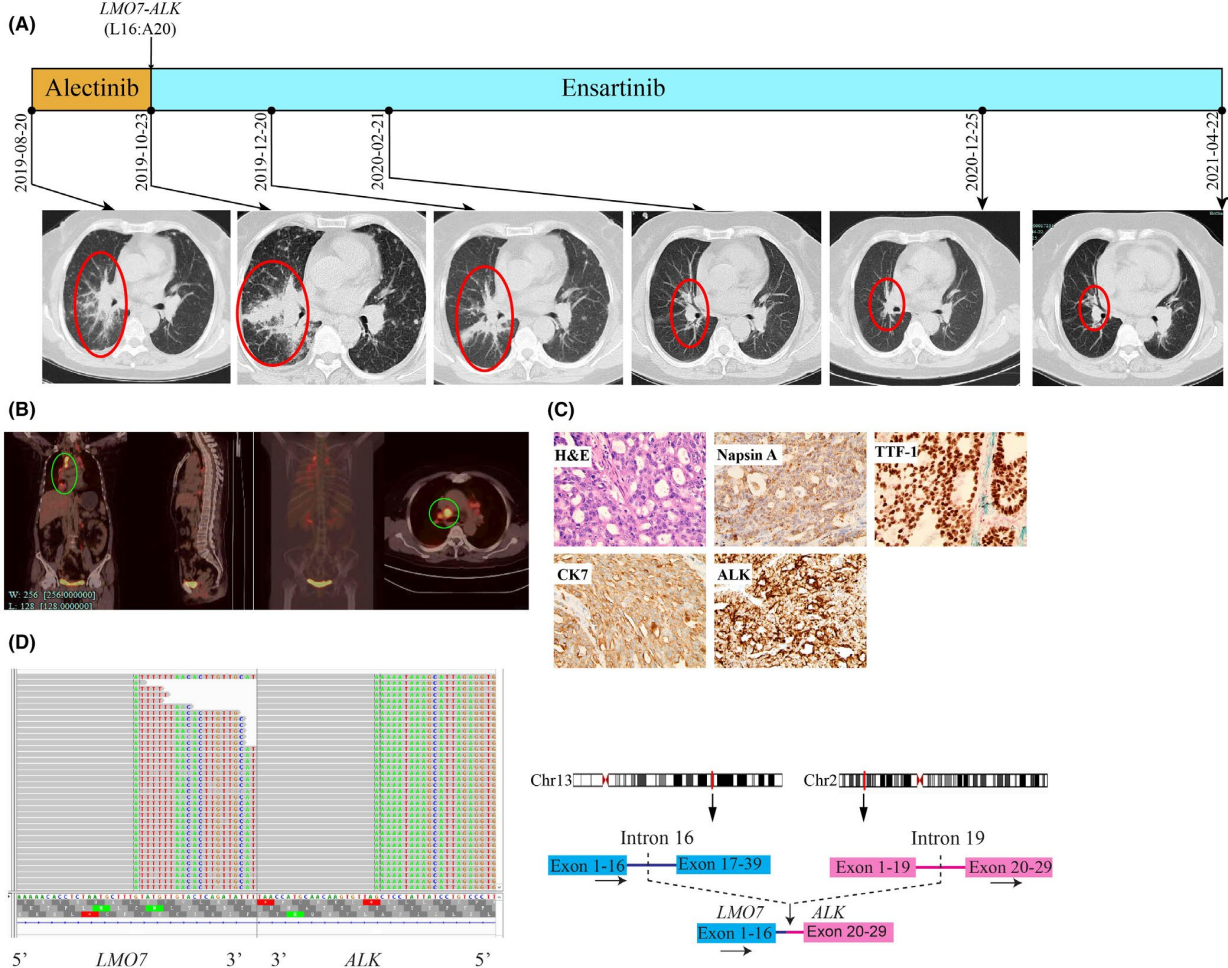
Schematic of treatment history and NGS‐detected *ALK* fusion of Case 2. (A) The timeline of treatment and CT scans are showed where the red circles highlight the location of tumours. The time point of NGS‐detected *LMO7*‐*ALK* is labelled above. (B) Baseline positron emission tomography (PET‐CT) scans with circled tumour location. (C) Pathological examination of the surgical specimen. The H&E staining image (400×) showed a poorly differentiated adenocarcinoma histology, and IHC testing results showed positive expression of Napsin A, TTF‐1, CK7 and ALK respectively. (D) Identification of the *LMO7*‐*ALK* fusion. Sequencing reads of *ALK* and *LMO7* are visualized by the Integrative Genomics Viewers (IGV, left). The schematic on the right shows the fused exons of the *LMO7*‐*ALK* rearrangement

A 56‐year‐old non‐smoking Chinese female patient was presented to our hospital with a chief complaint of chest tightness in August 2019. CT scans revealed a mass (17 mm × 9 mm) in the lower lobe of the right lung, multiple lung nodules and mediastinal lymph enlargement (Figure 2A). Further positron emission tomography‐computed tomography (PET‐CT) displayed multiple pulmonary metastases, multiple lymph node metastases and pleural involvement (Figure 2B). Bronchoscopic biopsy and IHC analysis revealed a poorly differentiated adenocarcinoma with positive expression of Napsin A, TTF‐1, CK7 and ALK (Ventana, Figure 2C). She was diagnosed as right lung adenocarcinoma (cT4N3M1a, stage IV) and treated with alectinib (600 mg, twice a day) as the first‐line treatment since August 2019. However, the disease progressed rapidly in two months as the follow‐up CT scans showed enlarged right hilum lesions (64 mm × 41 mm, Figure 2A). A second bronchoscopy was performed for targeted NGS analysis which detected a novel *LMO7*‐*ALK* fusion (Figure 2D). Subsequently, a second‐line ensartinib (225 mg daily) was prescribed since October 2019. Follow‐up CT scans showed a continuous shrinkage of the tumour, and PFS has exceeded 18 months till the last follow‐up visit on 22 April 2021.

## 3 DISCUSSION

Anaplastic lymphoma kinase fusion is a well‐studied carcinogenic alteration through the sustained ALK protein expression which then acts on the downstream signalling pathways. ALK‐TKIs work as antitumour agents by inhibiting the activation of ALK fusion protein.^2^ However, patients with diverse *ALK* fusions showed different responses to ALK‐TKIs as the 5’ *ALK* fusion partner could influence drug sensitivity by regulating the stability of fused protein.^3^ Furthermore, previous alectinib clinical trial was lack of the comprehensive information about *ALK* fusion partners^4^ which restricted the evaluation of alectinib efficacy in patients with rare *ALK* fusions.

In the first case, a *STRN*‐*ALK* fusion (S3:A20) was detected upon the first‐line alectinib treatment. The fused protein retained the coiled‐coil domain of STRN and the ALK kinase domain. This fusion could lead to constitutive activation of ALK kinase fusion via dimerization mediated by the coiled‐coil domain of STRN as proved in thyroid cancer both in vitro and in vivo.^5^ Previous studies showed aggressive features of tumours with *STRN*‐*ALK* fusion including lymph node metastasis and extraorgan extension.^5^, ^6^, ^7^ In lung cancer, a limited number of cases reported inconsistent responses to ALK‐TKIs as summarized in Table 2. The two patients treated with alectinib in front‐line showed extremely different responses: one progressed in three months and the other benefited for over 19 months. The alectinib nonresponder also carried the overexpression of *ABCB1* mRNA which was reported as an ALK‐TKI resistant mechanism.^8^ Thus, the concurrent alterations in these two patients might explain the different responses to alectinib. In contrast, the efficacy of crizotinib for patients with *STRN*‐*ALK* was promising in both first‐ and multiple‐line treatment. In our first case, taken together the positive ALK IHC result of pre‐alectinib sample and the NGS‐detected *STRN*‐*ALK* post‐alectinib, this rare *ALK* fusion is likely to be the primary driver mutation. However, whether the *MET* amplification was acquired during alectinib treatment is uncertain due to the lack of the NGS result of the pre‐alectinib sample. *MET* amplification is a recurrent resistant mechanism of ALK‐TKIs which was developed in 33% of *ALK*‐positive patients post second‐generation ALK‐TKIs.^9^ As the first case experienced a short partial response to alectinib, we tended to believe that the *MET* amplification was acquired and then led to the rapid progression. The correlation between *ALK* fusion and *MET* amplification was still less investigated, but another case reported a good response to crizotinib in an NSCLC patient harbouring *KLC1*‐*ALK* fusion and *MET* amplification.^10^ Similarly, in our first case, switching to crizotinib also led to a rapid and durable response.

**TABLE 2:** Reported ALK‐TKI responses in lung cancer with *STRN*‐*ALK* fusion

References	Year	Co‐alterations	ALK‐TKI	Line of treatment	Response
H. Ren et al.^15^	2019	Not mentioned	Crizotinib	1st‐line	PFS >4 years
Y. Yang et al.^16^	2017	*MYC* amplification; *TP53* (R181C)	Crizotinib	multiple	CR
C. Zhou et al.^17^	2019	*EGFR* (19DEL)	Crizotinib +gefitinib	3rd‐line	PR
Y. Nakanishi et al.^7^	2017	*ABCB1* mRNA overexpression	Alectinib	1st‐line	PD in 3 months
C. Su et al.^18^	2020	*GRM8* (E508K); *SETD2* (E1553K)	Alectinib	1st‐line	PR >19 months

Abbreviations: ALK, anaplastic lymphoma kinase; CR, complete response; PD, progressive disease; PFS, progression‐free survival; PR, partial response; TKI, tyrosine kinase inhibitor.

The *LMO7*‐*ALK* fusion (L16:A20) detected in the second case was a novel variant where the exon 16 of *LMO7* and the exon 20 of *ALK* were rearranged. LMO7, a multifunctional protein regulating actin cytoskeleton, assembly of adherens junctions in epithelial cells and gene expression,^11^ was first identified to be fused with v‐Raf murine sarcoma viral oncogene homolog B1 (BRAF) in papillary thyroid carcinoma and proved to increase extracellular signal‐regulated kinase (ERK) phosphorylation and promote anchorage‐independent cell growth.^12^ In lung cancer, a different *LMO7*‐*ALK* variant, L15:A20, was reported in molecular analysis of 158 *ALK*‐rearranged NSCLCs but no treatment information and clinical follow‐ups were available.^13^ Thus, we reported the *LMO7*‐*ALK* fusion with a novel breakpoint (L16:A20) and its poor response to alectinib for the first time. We chose ensartinib as the second‐line treatment which is a new‐generation ALK inhibitor with much higher potency than crizotinib. Ensartinib showed great efficacy in *ALK*‐positive patients regardless of previous TKI treatments. Even for those who had received crizotinib and other second‐generation ALK‐TKI, the disease control rate also reached 50% as reported in a phase I/II multicentre study of ensartinib in 2018.^14^ Notably, ensartinib displayed an encouraging efficacy on this rare type of ALK fusion in the second case who had progressed on the first‐line alectinib.

## 4 CONCLUSION

In conclusion, these two cases provided valuable clinical evidence of responses to ALK‐TKIs in patients with rare *ALK* fusions. Considering the inconsistent responses reported in previously published articles, the clinical efficacy of ALK‐TKIs including alectinib for lung cancer patients with uncommon *ALK* gene fusions remained to be evaluated with more cases and the comprehensive molecular profiling of tumours could assist precise treatment beyond canonical examinations such as IHC.

## CONFLICT OF INTEREST

Yutong Ma and Junrong Yan are the employees of Nanjing Geneseeq Technology Inc. Songan Chen is the employee of Burning Rock Biotech. The other authors report no conflicts of interest.

## AUTHOR CONTRIBUTION

**Mengnan Li:** Conceptualization (equal); Writing‐original draft (equal). **Zhou An:** Conceptualization (equal); Writing‐original draft (equal). **Qiusu Tang:** Conceptualization (equal); Data curation (equal). **Yutong Ma:** Formal analysis (equal); Software (equal). **Junrong Yan:** Formal analysis (equal); Software (equal). **Songan Chen:** Software (equal). **Yina Wang:** Conceptualization (equal); Supervision (lead); Validation (lead); Writing‐review & editing (lead).

## CONSENT FOR PUBLICATION

Written informed consent was obtained from the patients for publication of the case report and accompanying images.
